# Raman-Activated Cell Ejection for Validating the Reliability of the Raman Fingerprint Database of Foodborne Pathogens

**DOI:** 10.3390/foods13121886

**Published:** 2024-06-15

**Authors:** Shuaishuai Yan, Xinru Guo, Zheng Zong, Yang Li, Guoliang Li, Jianguo Xu, Chengni Jin, Qing Liu

**Affiliations:** 1 College of Food Science, Shanxi Normal University, Taiyuan 030031, China; yanshuaidouble@163.com (S.Y.); shine_gxr77@163.com (X.G.); qwasz4399@outlook.com (Z.Z.); 13834940755@163.com (Y.L.); 61254368@163.com (G.L.); xjg71@163.com (J.X.); 2 School of Health Science and Engineering, University of Shanghai for Science and Technology, Shanghai 200093, China; 3 School of Food and Biological Engineering, Shaanxi University of Science and Technology, Xi’an 710021, China

**Keywords:** foodborne pathogen, phenotype, Raman-activated cell ejection, sequencing, genotype

## Abstract

Raman spectroscopy for rapid identification of foodborne pathogens based on phenotype has attracted increasing attention, and the reliability of the Raman fingerprint database through genotypic determination is crucial. In the research, the classification model of four foodborne pathogens was established based on t-distributed stochastic neighbor embedding (t-SNE) and support vector machine (SVM); the recognition accuracy was 97.04%. The target bacteria named by the model were ejected through Raman-activated cell ejection (RACE), and then single-cell genomic DNA was amplified for species analysis. The accuracy of correct matches between the predicted phenotype and the actual genotype of the target cells was at least 83.3%. Furthermore, all anticipant sequencing results brought into correspondence with the species were predicted through the model. In sum, the Raman fingerprint database based on Raman spectroscopy combined with machine learning was reliable and promising in the field of rapid detection of foodborne pathogens.

## 1. Introduction

Foodborne diseases caused by the consumption of food and water infected with pathogens are one of the biggest challenges faced by human health [1,2]. Conventional microbial detection strategies involve the gold standard (biochemical analysis), nucleic acid-based assays (polymerase chain reaction, PCR) and immunological methods (enzyme-linked immunosorbent assay, ELISA), etc. These methods are time-consuming and laborious because they require a lengthy bacterial enrichment process [3,4]. Therefore, the development of more sensitive methods to rapidly diagnose pathogens at an early stage of contaminated food is essential to ensure food safety.

Raman spectroscopy with high spatial resolution and non-invasive capabilities can realize the detection of food-borne pathogens at a single-cell level without pre-enrichment of bacteria in food samples. Raman spectroscopy enables rapid acquisition of comprehensive information on nearly all chemical elements within a single cell in second [5]. The integration of these data is known as single-cell Raman spectra (SCRS), and is used to reveal the phenotypic characteristics and physiological metabolic differences among diverse microorganisms [6]. Furthermore, the combination of SCRS and appropriate machine learning methods can effectively overcome the spectral limitations caused by weak signals, low signal-to-noise ratios (SNR), complex information, and highly similar data [7]. Therefore, Raman spectroscopy has been successfully applied to the research of individual bacteria in the environment [8,9], clinic [10,11], food [12] and intestinal tract [13]. Much of the recent research demonstrated that the Raman fingerprint database can distinguish foodborne pathogens of different species, serotypes, growth cycles and nutritional statuses [14,15]. It is worth noting that the recognition program was based solely on artificial intelligence (AI) predictions and has not been further validated through biological strategies. To evaluate the effectiveness of the established database for recognizing foodborne pathogens in multiple pooled samples, the following procedures should be performed: (i) Phenotype analysis, and the classification models should be explored based on SCRS of different strains. (ii) Investigations into how to gain an interested single bacterium. (iii) After amplification of genomic DNA and sequencing of the target bacteria, judgement of whether the phenotype and genotype match.

The most crucial point in the above processes is the acquisition of marked single cells from complex microbial communities. Raman-activated cell ejection (RACE) based on the principle of laser-induced forward transfer (LIFT) can separate target bacteria from glass slides with aluminum layers by means of a pulsed laser, to overcome one of the most momentous parts of biological strategy. The procedure of LIFT was normally very fast on account of small objects being transferred through a pulsed laser. Furthermore, the heat generated by the pulse laser of appropriate power was extremely limited; it was almost harmless to cells [16]. So far, RACE has been applied to investigate the genome and metabolic mechanism of microorganisms from oral cavity [17], soil [18], ocean [19], intestinal tract [20] and so on. Therefore, RACE has tremendous untapped potential in the field of rapid identification for foodborne pathogens.

In this study, a Raman fingerprint database of diversiform foodborne pathogens was created. Predictions were made based on the database for stochastically selected single cells in the mixed bacterial sample. These marked cells were subsequently sorted one by one through RACE. The target bacteria for amplification of genomic DNA were designated prior to sequencing. Finally, the results of upstream prediction were compared with downstream sequencing to validate the accuracy of the discernment model, thus indicating that the database established is to be endowed with biological significance.

## 2. Material and Methods

### 2.1. Bacterial Culture and Sample Preparation

Four foodborne pathogens were obtained from the American Type Culture Collection (ATCC, Manassas, VA, USA). *Escherichia coli* O157:H7 (*E. coli* ATCC 43895), *Vibrio parahaemolyticus* (*V. parahaemolyticus*, ATCC 33847), *Listeria monocytogenes* (*L. monocytogenes*, ATCC 19115) and *Staphylococcus aureus* (*S. aureus*, ATCC 29213), stored in −80 °C and 25% glycerin were cultivated in a stationary growth phase at 37 °C in nutrient broth. Next, 1 mL of bacterial culture was centrifuged in a refrigerated centrifuge at 6000 rpm for 3 min; the bacterial sediment was washed with sterile deionized water at least three times to remove residual media and impurities after discarding the supernatant. The 2 μL of each specimen suspension at the appropriate concentration was pipetted onto the sorting chip and allowed to dry in a sterile operating table for the arrangement of capturing SCRS. For each sample, three separate lots were afforded.

### 2.2. Single-Cell Raman Spectra Acquisition

The chip involving the bacterial samples was located at the XYZ platform of the Raman spectroscopy system equipped with a 100× objective (P300, HOOKE Instruments Ltd., Changchun, China). The integration of a 532 nm neodymium-yttrium aluminum garnet (Nd:YAG) laser and 1200 groove/mm diffraction grating for receiving SCRS after silicon wafer standardization, and the air-cooled charge coupled detector (CCD) of −75 °C caused low signal-to-noise spectra to restore the original information of the sample. The laser power and irradiation time for each spectrum were 3 mW and 1 s, respectively, and only one cumulative acquisition was executed. All spectra ranges were distributed between 400 cm^−1^ and 2000 cm^−1^. For each strain, approximately 200 single cells were randomly selected for SCRS registration to construct subsequent classification models.

### 2.3. Data Preprocessing and Analysis

The standardized preprocessing of raw spectral data was the first critical routine for establishing an anticipant model, which cannot only calibrate physical interferences caused by sample thickness, experimental batches, random instrument noise and laser optical paths, but also weaken the influence of the signal generated by irrelevant chemical components. All original spectra obtained were subjected to a uniform preprocessing procedure, which comprises the elimination of abnormal high-intensity spectra, the subtraction of background signal, removal of cosmic rays, polynomial baseline correction, smoothing and normalization [21]. Then, in order to facilitate the analysis, management and generalization of the pretreatment data, dimensionality-reduction algorithms that can transform high-dimensional into low-dimensional data were the second critical routine for an ideal categorizer. As a nonlinear dimensionality-reduction approach, t-distributed stochastic neighbor embedding (t-SNE) can filtrate and extract the most representative characteristics from high-dimensional data with thousands of features, and is capable of guaranteeing the rationality of fitting results [22]. Three supervised arithmetics based on the scikit-learn algorithm package (Python, version 3.7.2) were utilized, involving support vector machine (SVM), K nearest neighbor (KNN) and linear discriminant analysis (LDA). The optimal classifier was yielded through 10-fold cross-validation, confusion matrix and receiver operating characteristic curve (ROC curve).

### 2.4. Identification and Ejection of Bacteria with Unknown Tags

**Figure 1:**
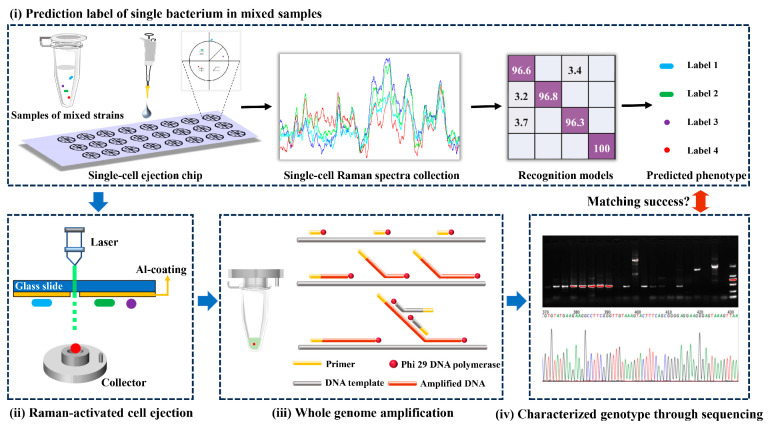
The scheme of validation for Raman fingerprint database of foodborne pathogens based on RACE.

Equal amounts of four washed foodborne pathogen suspensions were mixed in a sterile EP tube and vortexed thoroughly. A 2 μL amount of multicomponent sample was placed at the specific position of the sorting chip, and then air-dried in a laminar airflow chamber. The aluminum-coated single-cell ejection chip resembled a two-dimensional rectangular coordinate and was divided into four regions, each of which was marked with a special shape to facilitate the localization of the target bacteria (Figure 1(i)). The Raman spectrum of each randomly picked cell was then recorded, and the SCRS of these unknown groups was recognized by well-established classifiers, while the coordinates of the above familiarized cells were registered for further sorting [23].

The chip was inverted and immobilized on a single-cell separation device equipped with a 532 nm Nd:YAG laser, 10× objective and CCD imaging system (PRECI SCS, HOOKE Instruments Ltd., Beijing, China). Based on the marked coordinates, the laser pulse capable of passing through the transparent glass substrate of the chip was focused on the coating to vaporize the layer (Figure 1(ii)); 10 cells identified as the same label were completely sorted into a collector already filled with cell lysis buffer (Qiagen, Hilden, Germany). Five groups were repeated for each strain.

### 2.5. Amplification of Genomic DNA and Sequencing

Using a REPLI-g Single Cell Kit (Qiagen, Germany), the femtogram-level DNA in the collector was high-quality amplified to a microgram level for sequencing through multiple displacement amplification (MDA) [23]. Briefly following the procedure of the kit (Figure 1(iii)), all collectors arranged in sterile petri dishes were repeatedly frozen and thawed at −80 °C to accomplish as much lysis as possible of the target bacteria. Adaptable collectors were docked to PCR tubes and centrifuged to harvest target bacteria containing lysates. The PCR tubes were heated in a thermal cycler at 65 °C for 10 min. A 3 μL amount of stop solution and 40 μL of crucial operating solution (including reaction buffer and DNA polymerase) were added to the tubes. The commixture was constantly incubated at 30 °C for 8 h before inactivating DNA polymerase at 65 °C for 3 min. Amplification products of genomic DNA were stored at −20 °C for further downstream sequencing.

The quality of amplification products was appraised by PCR and visualized agarose gels. Two pairs of universal primers involved 27F (5′-AGAGTTTGATCCTGGCTCAG-3′)/1429R (5′-TACGGCTACCTTGTTACGACTT-3′) and 341F (5′-ACTCCTACGGGAGGCAGCAG-3′)/806R (5′-GGACTAVHVGGGTWTCTAAT-3′) for the bacterial 16S rRNA gene amplification. Amplification products with specific visible bands in agarose gels were subjected to Sanger sequencing (Sangon Biotech Co., Ltd., Shanghai, China) and Illumina sequencing, respectively. DNA sequences obtained from the former were matched through Blast of NCBI, while the latter was based on the Illumina MiSeq PE300 platform (Majorbio Bio-Pharm Technology Co., Ltd., Shanghai, China) for microbial diversity analysis (Figure 1(iv)) [24]. The sequence was stored in FASTQ format. De-hybridized double-ended sequences were preprocessed using FLASH software (Version 1.2.11). The aligned reads were clustered into operational taxonomic units (OTUs) by Usearch (Version 11) with a sequence similarity threshold of ≥97%. Based on the Silva 16S rRNA database, taxonomic information of the OTUs was undertaken by the Quantitative Insights Into Microbial Ecology (QIIME) software (version 1.9.1). According to the results of taxonomic analysis, the species composition of different samples at different taxonomic levels was obtained to reveal the genotype of unknown target bacteria.

The products without specific visible bands were subjected to metagenomics sequencing [25]. Briefly, genomic DNA samples were sheared into 400–500 bp fragments using a Covaris M220 (Gene Company Limited, Hong Kong, China). Illumina sequencing libraries were prepared using NEXTFLEX™ Rapid DNA-Seq (Bioo Scientific, Austin, TX, USA). Paired-end DNA sequencing was performed on the Illumina Novaseq6000 (Illumina, San Diego, CA, USA) platform at the Majorbio (Majorbio Bio-Pharm Technology Co., Ltd., China).

## 3. Results and Discussion

### 3.1. SCRS of Foodborne Pathogens

**Figure 2:**
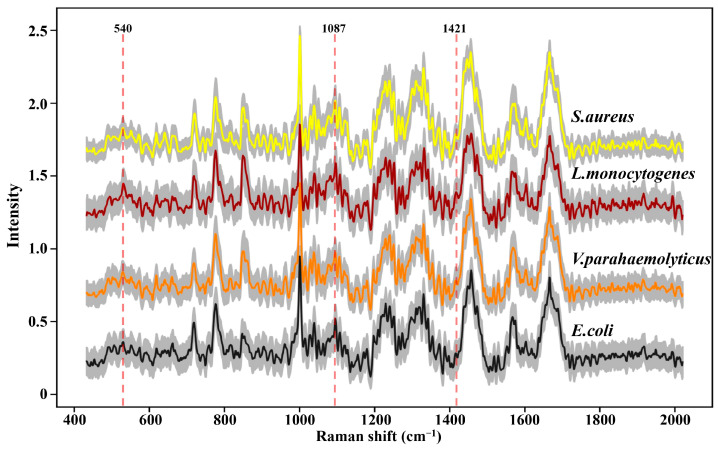
Raman spectra of four foodborne pathogens. At least 200 single-cell Raman spectra were acquired for each strain. The colored thick solid lines represent the average Raman spectra; the gray area represents standard deviation.

SCRS with the thousands of peaks and valleys was a visual representation that revealed the molecular fingerprints of the chemical constituents from an intact cell. In order to escape potential laser-induced damage to bacteria as much as possible, 200 SCRS of each strain were, respectively, captured by dint of weaker laser power and shorter exposure time. The conspicuous painted lines in Figure 2 manifested the average SCRS of the four strains, and the major spectral signatures between multifarious species were extremely similar; it was almost impossible to distinguish these spectra through observation. Furthermore, the milder acquisition conditions observably reduced the SNR of SCRS (distributed between 3 and 6 in Figure S1), which generated more small thorns and noise, and enhanced the difficulty of spectral discernment.

**Figure 3:**
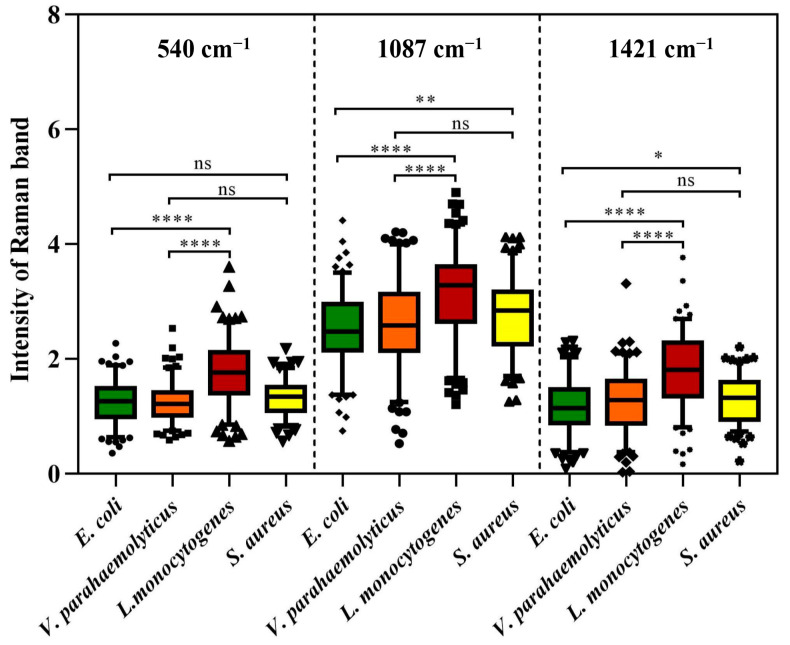
Raman intensity of four foodborne pathogens at peaks 540 cm^−1^, 1087 cm^−1^ and 1421 cm^−1^. Box plots represent the median and first and third quartiles, with the whiskers representing the minimum and maximum values within 1.5 interquartile ranges from the first and third quartiles. Black dots with different shapes indicate outliers. Two-tailed *t*-tests were used to compare the statistical significances. ns *p* > 0.05, * *p* ≤ 0.05, ** *p* ≤ 0.01, **** *p* ≤ 0.0001.

However, as an aggregation of different molecular vibration information, each band in SCRS has a corresponding molecular assignment decipherment, which can be applied to find out the potential reasons for the deviations between different species through statistical analysis. As we all know, the cell walls of Gram-positive bacteria (G^+^) contain more peptidoglycan and teichoic acid than those of Gram-negative bacteria (G^−^). Previous research has demonstrated that 540 cm^−1^ and 1421 cm^−1^ were assigned to the visible peaks of peptidoglycan, and 1087 cm^−1^ was assigned to the typical band of teichoic acid (Figure 2) [26]. As shown in Figure 3, the Raman response values of four foodborne pathogens at 540 cm^−1^, 1087 cm^−1^ and 1421 cm^−1^ were statistically analyzed and *t*-test was performed. Regardless of which of the three peaks was addressed, G^+^ exhibited stronger intensities compared to G^−^. *L. monocytogenes* revealed obvious spectral signals contrasted with both of the G^−^, while *S. aureus* exhibited significant intensities at 1087 cm^−1^ and 1421 cm^−1^ compared to *E. coli*, which was consistent with previous research findings [24]. Specific peak analysis may capacitate the authentication of G^+^ and G^−^, but for investigation of finer distinctions, it is necessary to filter the mutual characteristics among different strains and excavate subtle difference features for the discernment of foodborne pathogens through AI algorithms [27].

### 3.2. Classification Models for Recognition of Foodborne Pathogens

**Figure 4:**
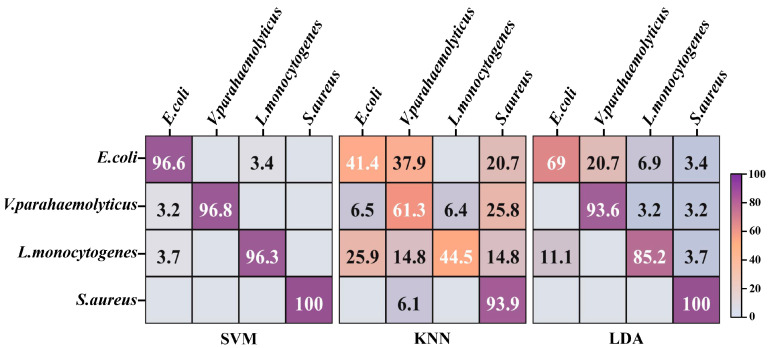
The confusion matrix of four foodborne pathogens based on machine learning models. SVM: support vector machine; KNN: K nearest neighbor; LDA: linear discriminant analysis.

It is critical to investigate the most suitable one for divination of foodborne pathogens among a wide range of machine learning approaches. The t-SNE algorithm was devoted to nonlinearly mapping the high-dimensional data of 800 spectra from the 4 strains into the low-dimensional space, so that the global and internal structure information of critical data in the low-dimensional space was as similar as possible to the data features in the high-dimensional space. The tactic of 10-fold cross validation, which can evaluate the ability of classification systems to predict new datasets, was applied to assess the categorization performances of SVM, KNN and LDA. Larger numbers and darker purple exhibited higher distinction accuracy on the diagonal in the confusion matrix (Figure 4). The highest identification accuracy of SVM for 4 strains was 97.43%, which surpassed the recognition accuracy of 86.23% for 23 strains in our previous research [12]. There was a slight misrecognition between *E. coli* and *L. monocytogenes* in the confusion matrix of SVM, which was potentially attributed to their morphological similarities. The accuracies of LDA and KNN for the identification of 4 strains were 86.95% and 60.28%, respectively. Both categorizers misclassified numerous *E. coli* as *V. parahaemolyticus*, which may be related to their analogous composition derived from G^−^. KNN displayed diminished performance that erroneously discerned plentiful *V. parahaemolyticus* and *L. monocytogenes* as other strains, which can probably be ascribed to the lack of loss function for feature weight self-adjustment [28]. In addition, the values of micro-average, macro-average and sample dimension in the ROC curves of SVM were approximate to 1, higher than those values in the ROC curves of KNN and LDA (Figure S2). This evidenced that SVM was equipped with the optimal performance for further prediction of unknown strains in multiple pooled samples.

### 3.3. Examination of Single Cells’ Sorting Efficiency

To ensure that each sorted single cell was accurately received by the receptor, the ejection efficiency of the sorter, the receiving usefulness of the collector and the stability of the instrument were executed in detail. *E. coli*, as the patterned strain, was distributed on the chip, and 50 individually dispersed *E. coli* were sorted into the identical receiving unit each time (Figure S3a). Firstly, the detachments of the interested *E. coli* at the ejection chip locations were observed to appraise the sorting efficiency (Figure S3b). Subsequently, the number of sorted *E. coli* contained in the receiver was enumerated under a microscope to inquire into the receiving efficiency (Figure S3c,d). Finally, the constancy of the entire procedure was validated by multiple dates.

**Figure 5:**
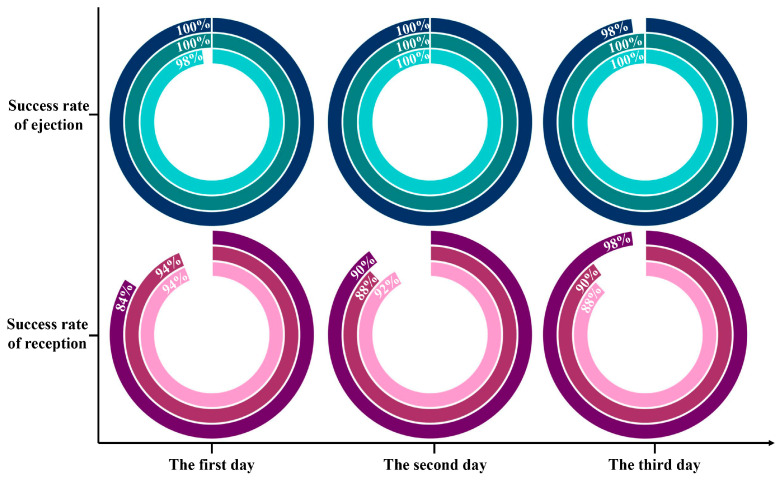
Statistics on the success rates of ejection and reception for target bacteria based on RACE. Each ring is an individual test.

Based on plenty of repeated verifications, the success rates of ejection and reception were 99.56 ± 0.88% and 90.88 ± 4.13%, respectively. Figure 5 manifested that single cells captured from the chip had excellent reproducibility and competence, which probably gave the credit to the appropriate sorting laser and effortless vaporization of the thin layer. Notably, the lowest success rate for receiving was only 84%, indicating that there was still a possibility of not collecting the target single cells. On the one hand, ocular calculation of individual bacteria in the receiver perhaps omitted some unimpressive cells. On the other hand, even though the distance between the collector and the sorting chip was extremely tiny, it is possible that the target bacteria were indeed not launched into the receiver due to the disturbance of the air flow. Therefore, despite having the upper hand in arresting the target single cells, the absolute cleanliness and steadiness of the implement circumstances were guaranteed as far as possible during the single-cell sorting operation to prevent the negligence of bacteria of interest.

### 3.4. Recognition of Target Bacteria through RACE

The single cells with unknown species in the impure sample were investigated according to the procedure shown in Figure 1. Four types of foodborne pathogens were blended at equal magnitudes and dispersed on a sorting chip (Figure S4), in which an individual bacterium was randomly checked to collect SCRS for species prediction. Based on the constructed recognition model, the pathogens assigned labels were sorted for amplification of genomic DNA, and then genome sequencing was performed to judge whether the pathogens identified through AI model were correct. The specific bright bands in the gel electrophoresis images of 16S rRNA PCR using 27F and 1492R primers are shown in Figure S5, which complied with the requirements of Sanger sequencing for strain identification. The desired bright bands appeared in only 12 out of 20 groups. For one thing, this may involve the incomplete DNA of the target bacteria due to the damage caused by the laser to single cells. For another thing, it may also be related to the amplification bias during the MDA. Concerning the 12 bright bands including 4 from *E. coli*, 4 from *V. parahaemolyticus*, 3 from *L. monocytogenes* and 1 from *S. aureus*, the PCR amplification success rates of the genome from G^−^ were much higher than those of G^+^. G^−^ involving more peptidoglycans and teichoic acid in the cell wall was more resistant to laser exposure than G^+^, which may be associated with the resistance mechanism of G^+^ and G^−^ to antibiotics and fungicides [29,30]. Moreover, Sanger sequencing was used to acquire the sequences of 12 amplified products, which were searched through NCBI to obtain the practical labels of the strains for alignment with previous predicted species. Ten among the 12 groups were matched correctly (Table 1), and the accuracy was 83.3% in the validation of upstream prediction and downstream sequencing, among which the comparison accuracies of *E. coli*, *V. parahaemolyticus* and *S. aureus* were 100%. Two of the three groups from *L. monocytogenes* mismatched, and the sequencing results revealed *Micrococcus luteus* and *Cutibacterium acnes*, respectively. This may be closely related to contamination from external sources during the single-cell amplification, as these microorganisms are frequently present on the skin surface and in the air [31].

**Figure 6:**
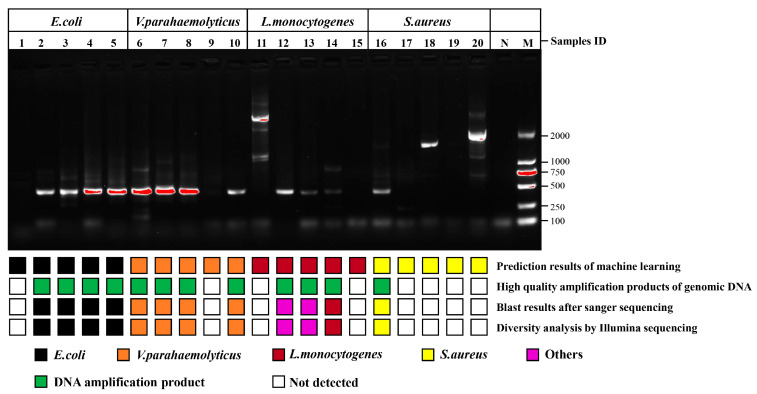
Comparison of upstream predicted phenotypes based on classification model and downstream genotypes based on genome sequencing. High-quality amplification products of genomic DNA represent the specific bright bands in the gel electrophoresis images of 16S rRNA PCR products from post-RACE cells using primers pair 341F and 806R. Lanes 1 to 5 were *E. coli*, lanes 6 to 10 were *V. parahaemolyticus*, lanes 11 to 15 were *L. monocytogenes*, lanes 16 to 20 were *S. aureus*, lane N and lane M were negative control and marker, respectively.

Since each collector contained 10 bacteria predicted to be of the same species, the few proportions of non-target DNA may not be exhibited by Sanger sequencing due to the existence of rare prediction errors, and the detailed species populations of the amplified genome were resolved through diversity analysis of Illumina sequencing. Sample groups showing specific bright bands in the gel electrophoresis images of 16S rRNA PCR using 341F and 806R primers were completely consistent with 16S rRNA PCR using the full-length primers, indicating that the genomes of eight groups without bright bands were damaged (Figure 6). The diversity analysis consequences were exactly the same as the Sanger sequencing (Figure S6 and Table 1), and the phenotypes forecasted by the algorithm were identical to the genotypes through sequencing. These results displayed that the approach of sequencing after RACE was feasible for the genetic dissection of target bacteria in complex communities. Notably, the abundances of the two mismatched samples including 12 and 13 were uncorrelated with the original species, which might be attributed to exogenous contamination during MDA. *Cutibacterium acnes*, *Micrococcus luteus* and *Malassezia* are usually considered normal inhabitants of human skin. Studies have shown that these organisms were present in the dermis of their skin from patients undergoing shoulder surgery despite strict and standard disinfection measures, including epidermal alcohol rubbing and intravenous antibiotic administration [32,33]. Although single-cell sorting and MDA were completed in the sterile laminar flow chamber and thoroughly treated with 75% alcohol and RNase removal spray, they was still affected by environmental and human microbiota when the collector was removed due to the highly sensitive DNA amplification pathway. MDA strategies are highly sensitive to contamination due to the low DNA amounts of individual bacteria. Pre-sterilization of the reagent does not protect against endogenous/exogenous contaminants, which become more amplified in larger MDA reaction volumes due to reduced polymerase specificity. Performing MDA methods with smaller reaction volumes may be an effective way to reduce amplification bias and contamination [34].

**Figure 7:**
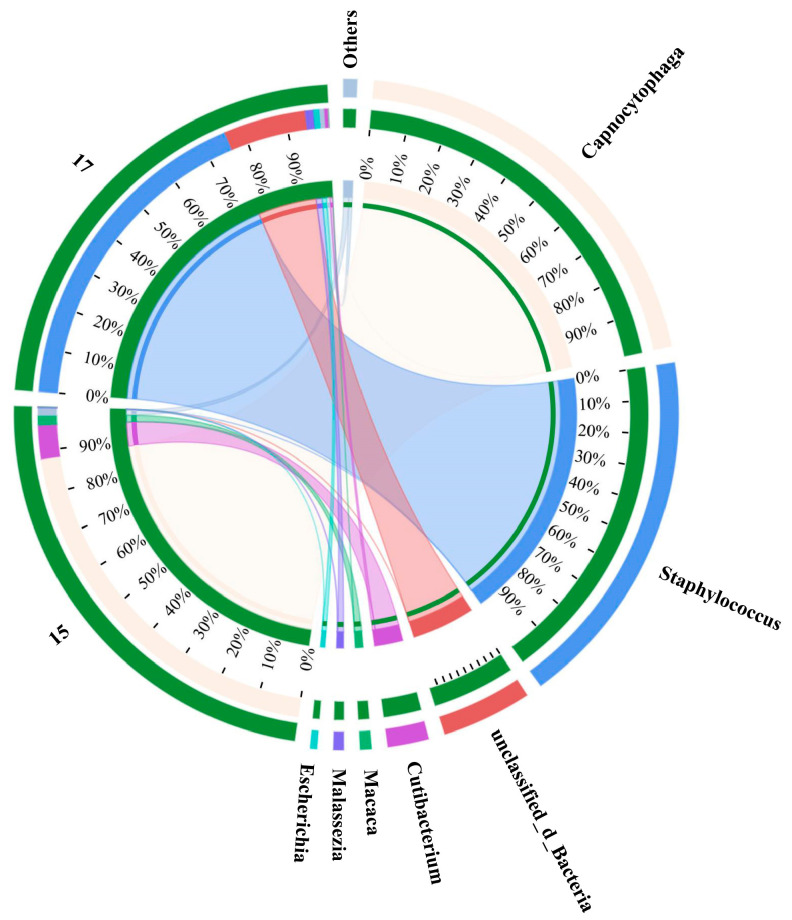
Distribution analysis of species in samples 15 and 17. Sample 15 was *L. monocytogenes* and sample 17 was *S. aureus*. The right side of the circle represents the main dominant species contained in each sample, and the abundance distribution of different species in the sample is shown by the connection of the inner ribbon.

Although lasers play an important role in advanced biological imaging and Raman spectroscopy, their widespread application was restrained on account of destructive effects on living organisms [35]. Whether SCRS collection or sorting of single cells, the laser beam used will be focused on the target cells, which greatly increases the risk of damage to the research object. A sperm cell membrane could be damaged under the irradiation at 30 mW laser power, but when the laser power is less than 15 mW, the chemical fingerprint information of a single live human sperm can be obtained [36]. With a laser power of 3 mW and acquisition time of 10 s, a collector containing five *E. coli* could generate matching sequencing results [37]. Therefore, the phenomenon of PCR gel electrophoresis images lacking expected bright bands under milder conditions still existed in the research. The comprehensive microbial communities of these false negative samples, especially G^+^, were further explored through metagenome sequencing to find the inherent factors (Figure 7). Although sample 15 represented *L. monocytogenes*, the actual microflora certified by metagenomic sequencing was comprised of 88% *Capnocytophaga*, 8% *Cutibacterium*, 2% *Macaca* and 2% others. As one of the thousands of resident microbial communities in the oral cavity, the relatively high proportions of *Capnocytophaga* in the sample manifested that the genome was observably contaminated [38]. *S. aureus* and the other three strains accounted for 76% and 0% of sample 17, respectively, proving that the classification model did not have misprediction. While there were no bright bands in the PCR gel electrophoresis images of the two pairs of primers in some groups, it is possible that the false-negative samples supported the predictions of the discernment model. Therefore, the alignment accuracy of upstream prediction and downstream sequencing was at least 83.3%, and the identification of foodborne pathogens by Raman spectroscopy based on machine learning was reliable.

Raman spectroscopy has great potential for rapid detection of foodborne pathogens; optimizing laser power and irradiation time may be an effective strategy for deciphering single cells based on RACE. However, the weakening of the SCRS collection conditions signified that the SNR of the obtained spectra was low, and the Raman features would become less distinct, which was unfavorable for the establishment of classification models and the discernment of target single cells in complex communities. In the future, the structure of the sorting chip will be redesigned and more appropriate pathways will be invented to decrease laser side-effects for individual cells [39,40,41]. Furthermore, the optimized amplification scheme of genomic DNA from single bacteria can be used to reduce the significant non-specificity and bias that often exist during amplification, and improve the amplification efficiency and coverage of the genome [37].

## 4. Conclusions

In summary, the single-cell phenotype and genotype were linked employing RACE, and the constructed Raman fingerprint database of foodborne pathogens was verified through single-cell genomic DNA amplification and sequencing. The classification model of four foodborne pathogens was established based on the t-SNE-SVM algorithm. The target bacteria named by the model were sorted through RACE. The predicted phenotypes were consistent with the results of single-cell genome sequencing, which demonstrated that the Raman fingerprint database was promising for the detection of foodborne pathogens at the single-cell level. Moreover, rapid, non-destructive and highly sensitive Raman spectroscopy has the potential to be applied to the rapid diagnosis of foodborne pathogens in food contamination to ensure food safety.

## Figures and Tables

**Table 1:** Comparison of upstream prediction and downstream sequencing of single cells sorted from 12 groups from four foodborne pathogens.

Samples	Prediction Results of Machine Learning	Blast Sequence after Sanger Sequencing	Diversity Analysis by Illumina Sequencing
2	*E. coli*	*E. coli*	*Escherichia*
3	*E. coli*	*E. coli*	*Escherichia*
4	*E. coli*	*E. coli*	*Escherichia*
5	*E. coli*	*E.coli*	*Escherichia*
6	*V. parahaemolyticus*	*V. parahaemolyticus*	*Vibrio*
7	*V. parahaemolyticus*	*V. parahaemolyticus*	*Vibrio*
8	*V. parahaemolyticus*	*V. parahaemolyticus*	*Vibrio*
10	*V. parahaemolyticus*	*V. parahaemolyticus*	*Vibrio*
12	*L. monocytogenes*	*Micrococcus luteus*	*Micrococcus*
13	*L. monocytogenes*	*Cutibacterium acnes*	*Cutibacterium*
14	*L. monocytogenes*	*L. monocytogenes*	*Listeria*
16	*S. aureus*	*S. aureus*	*Staphylococcus*

## Data Availability

The original contributions presented in the study are included in the article/Supplementary Materials, further inquiries can be directed to the corresponding authors.

## Supplementary Materials

The following supporting information can be downloaded at: https://www.mdpi.com/article/10.3390/foods13121886/s1. Figure S1. Signal-to-noise ratio of single-cell Raman spectra from four foodborne pathogens; Figure S2. The area under the curve of the ROC displaying the reliability of the three machine learning models in distinguishing each strain; Figure S3. Examination of ejection efficiency for target single cells based on Raman-activated cell ejection (RACE); Figure S4. Morphology of different strains on sorting chip; Figure S5: The gel electrophoresis images of 16S rRNA PCR products from post-RACE cells using primers pair 27F and 1492R; Figure S6. Bacterial community of high-quality amplification products of genomic DNA at the genus levels.
