# Supplementary material for: Raman-Activated Cell Ejection for Validating the Reliability of the Raman Fingerprint Database of Foodborne Pathogens
Source: Foods. 2024 Jun 15;13(12):1886. doi: 10.3390/foods13121886 (PMC11203195; doi:10.3390/foods13121886)
Supplement: Supplementary file 1 [file foods-13-01886-s001.zip › foods-3012028-supplementary.pdf]

## Supplementary Materials

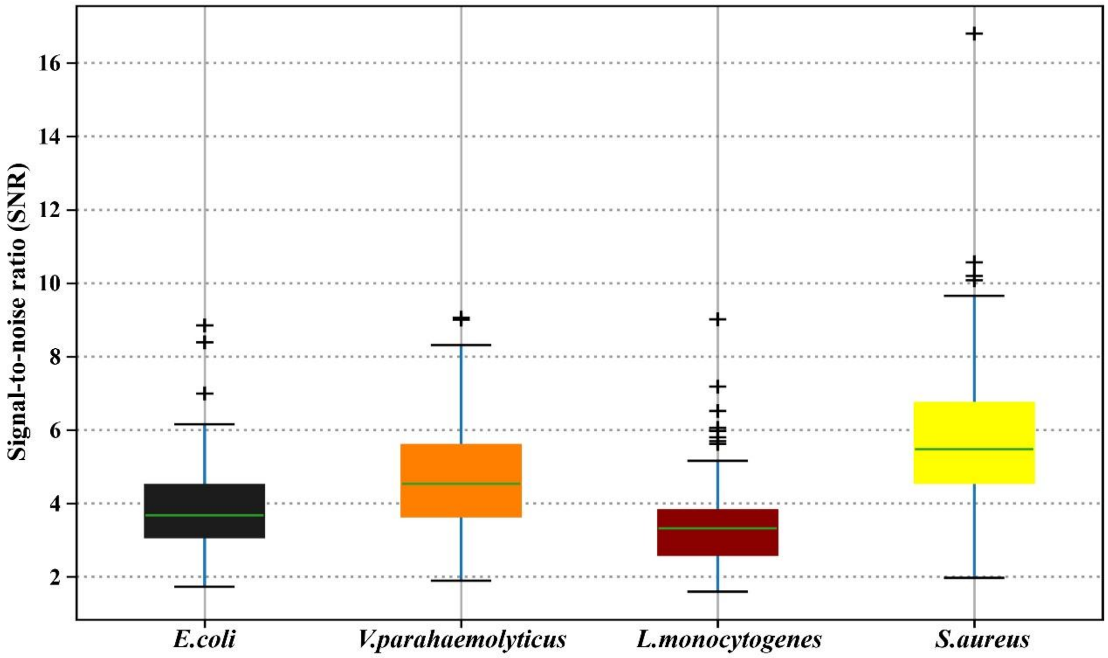


**Figure S1.** Signal-to-noise ratio of single-cell Raman spectra from four foodborne pathogens. At least 200 single-cell Raman spectra were acquired for each strain.

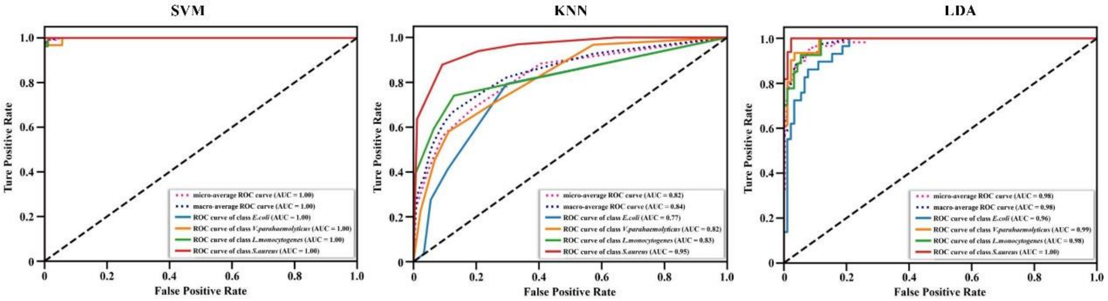


**Figure S2.** The area under curve of the ROC displaying the reliability of the three machine learning models in distinguishing each strain. SVM: support vector machine, KNN: K nearest neighbor, LDA: linear discriminant analysis.

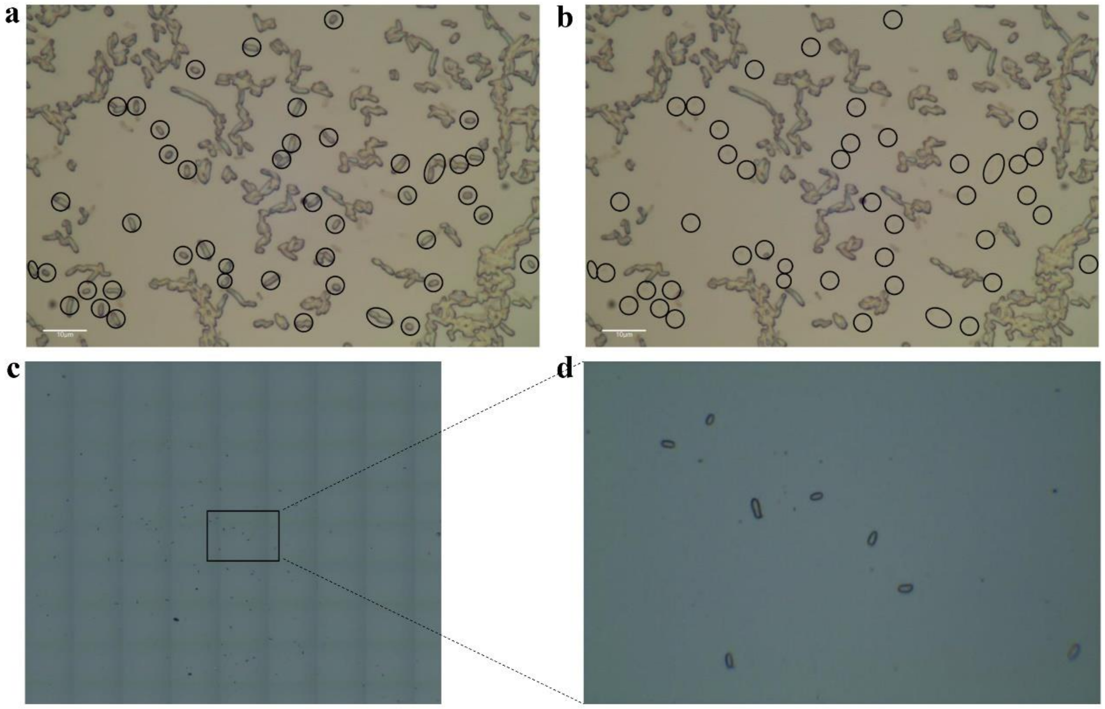

**Figure S3.** Examination of ejection efficiency for target single cells based on Raman-activated cell ejection (RACE). Black circles mark target single cells on sorting chips (a), single cell shedding after RACE (b), sorted single cells contained in the collector (c), local enlargement of the collector (d).

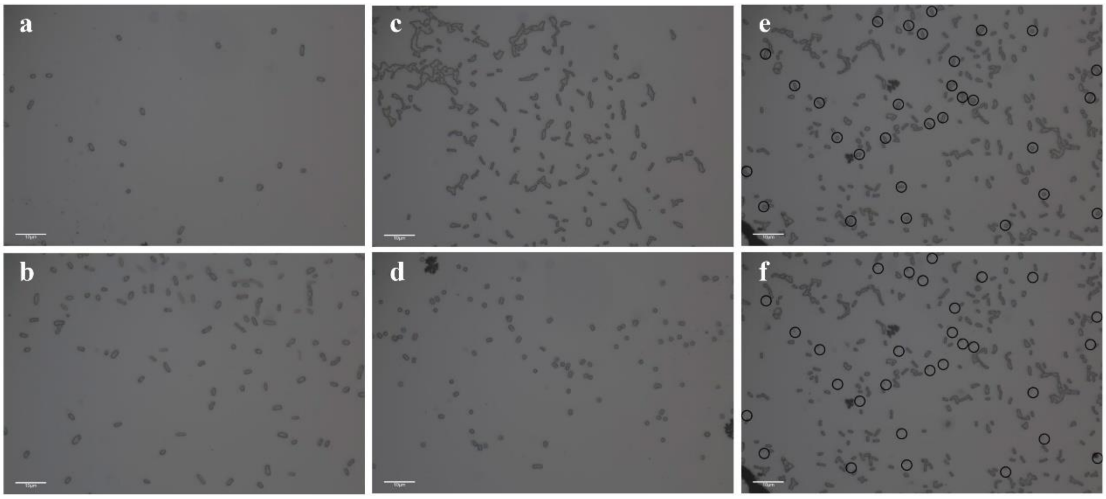

**Figure S4.** Morphology of different strains on sorting chip. (a) *E. coli*, (b) *V. parahaemolyticus*, (c) *L. monocytogenes*, (d) *S. aureus*, (e) mixed sample of four foodborne pathogens, (f) mixed samples after RACE sorting.

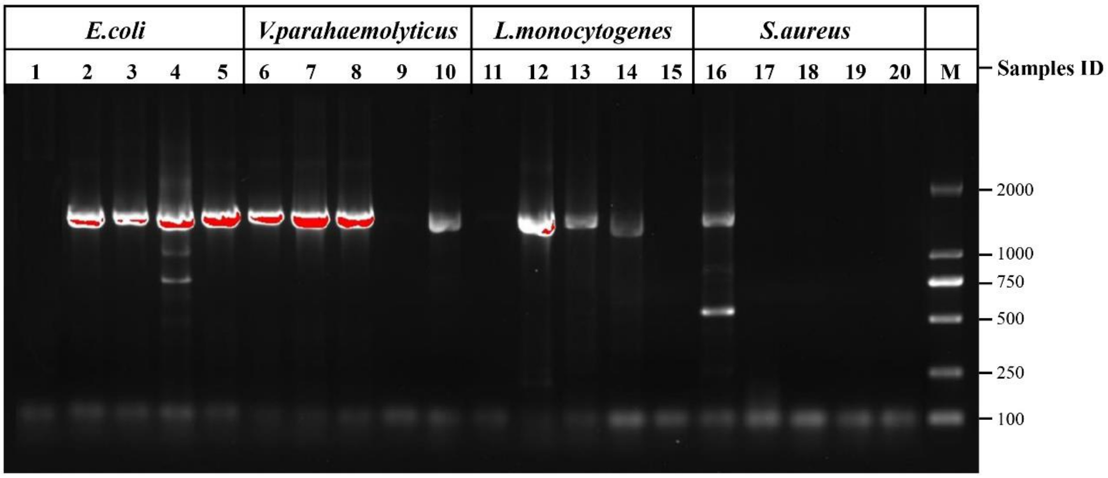


**Figure S5.** The gel electrophoresis images of 16S rRNA PCR products from post-RACE cells using primers pair 27F and 1492R. Lane 1 to 5 were *E. coli*, lane 6 to 10 were *V. parahaemolyticus*, lane 11 to 15 were *L. monocytogenes*, lane 16 to 20 were *S. aureus*, lane M was marker.

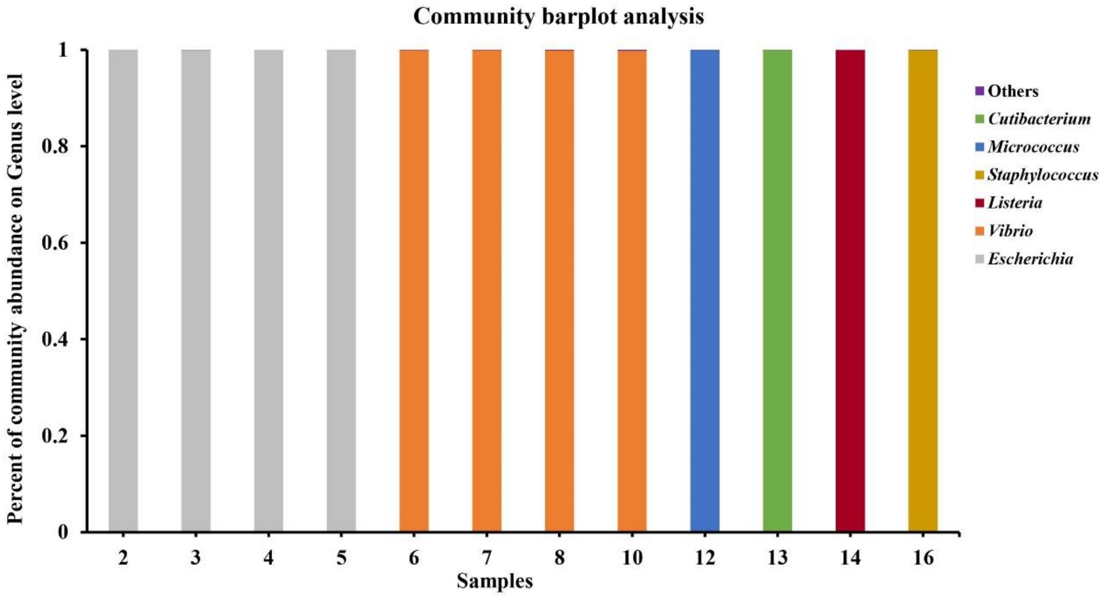


**Figure S6.** Bacterial community of high-quality whole genome amplification (WGA) products at the genus levels. High-quality WGA products represent that the specific bright bands in the gel electrophoresis images of 16S rRNA PCR products from post-RACE cells using primers pair 341F and 806R.
